# M. Delpech on Cancerous Tumours of the Jaws

**Published:** 1830-10-01

**Authors:** 


### XLVI.

### MONTPELLIER HOSPITAL.

CONSIDERATIONS ON CANCEROUS TUMOURS OF THE JAWS. By M. DELPECH.*

M. DELPECH, the able Professor of surgery in the hospital of Montpellier, has published some judicious observations on fungous tumours of the jaws, in the Journal of Medicine which he conducts.

M. Delpech commences by deploring the laxity with which the term polypus has been employed. Belonging, par excellence, to that vesicular formation which grows from the Schneiderian membrane, it has been extended to many other tumours existing in the nasal fossæ, the antra, and even the throat. After considering attentively the structure of the common vesicular polypus of the nose, and remarking that the periosteum beneath it is sometimes separated by effusion from the bone, or the latter altered in various ways, our author assumes that the polypus itself may be merely the consequence of this

* Mémorial des Hôpitaux du Midi, No. 16.

affection of the bone or periosteum. In support of his opinion he instances the fact which most surgeons, we believe, have remarked, that the polypus is more effectually cured, if a portion of the turbinated bone from which it grows is torn away at the same time. The following case is looked upon as a proof, that causes unconnected immediately with the mucous membrane may give rise to vesicular polypus.

*Case* 1. A robust man, a cook, experienced, at the age of fifty, pain in the frontal region, in the situation of the upper canine tooth on the right side, snuffling through the nose, sneezing, increased secretion from the nose, and frequent hæmorrhage. In the right nasal fossa was a tumour having all the characters of vesicular polypus, which was torn away without bleeding being the consequence. The eye was now observed to be unnaturally prominent, and the bones of the nose to be slightly twisted to the right. In a short time the nasal fossa filled again, the septum was so pushed to the left as to obliterate the cavity on that side, the eye was thrust out completely and vision destroyed, the palatine arch was pushed down, the teeth fell out, and a tumour usurped the place of the right canine fossa. This tumour was at first hard, but soon became soft, fluctuating, and pulsated distinctly. The pulsation extended to the other portions of the tumour in the mouth and nostril, and was even communicated to the eye. On making pressure with the finger the whizzing, so characteristic of aneurism, was felt deeply seated in the tumour. The pulsation ceased on compressing the carotid artery, and reappeared when that pressure was withdrawn. The growth of the tumour was now become enormous, and the hæmorrhage from the nose was frequent and alarming. In less than a month from this time he died worn out by the fever and irritation.

On dissection an aneurism was found to have sprung from the internal maxillary artery, immediately before the origin of the posterior palatine and spheno-palatine branches. It had destroyed the posterior wall of the antrum which it filled, and had also opened into the orbit.

M. Delpech believes that the vesicular polypus was merely a symptom of the pressure exercised on the periosteum and mucous membrane, and of the obstruction offered to the circulation in those textures. He has published a case of a similar kind in the Révue Medicale. The tumour was formed by the enlargement of the branches of the internal maxillary, and penetrated into the nasal fossa, the orbit, the mouth, and the substance of the cheek. There was here likewise a vesicular polypus, but the pulsations of the tumour pointing out its nature, M. Delpech tied the common carotid artery, when the polypus disappeared, though the aneurism was not cured. It is curious certainly that a polypus should be combined with the other tumour in both these cases, but we cannot assert with confidence that they were wholly and solely cause and effect.

The malignant tumours which form in the substance of the maxillary bones, and extend into all the neighbouring cavities, are considered as polypi, and distinguished when they project into the throat, by the epithets fibrous and fleshy. We do not believe that the term polypus is so lavishly employed in this country, as it seems to be in France, but perhaps even here it is applied too indiscriminately, to malignant and other tumours about the nose, and contiguous passages. M. Delpech considers it highly important to be aware, that these tumours most frequently have their origin in the neurilema of one of the nerves of the affected part. For proof of this position, he refers to the toothache, with which the disease occasionally commences, when one or more of the teeth falling out or being removed, the fungus fills their place and rapidly extends into the cavity of the mouth. The next case is offered in support of this opinion.

*Case* 2. A child, ten years old, was brought to M. Delpech with the left side of the face enormously enlarged by a tumour, which had driven out the upper teeth, augmented the superior maxillary bone to six times its natural size, thrust downwards the palatine arch, filled the nasal fossa, made its way into the orbit, and pushed out the eye. A vesicular polypus existed in the nostril. The disease had commenced with pains in the last molar teeth, which, with the remainder on that side had fallen out, when a medullary tumour from the alveoli forced its way into the mouth. M. Delpech operated by laying bare the maxillary bone and opening into the cavity of the mouth, and then removing the anterior wall of the maxillary sinus and the whole of the alveoli. The disease was found to spring from the posterior wall of the sinus, that part where the vessels and superior-posterior dental nerves pass into the bone. By the fingers, cutting instruments, and the actual cautery, the tumour was followed up with care but determination. It had pierced the anterior wall of the foramina by which the posterior palatine canal communicates with the posterior alveoli, and M. Delpech was obliged to break these up more extensively. The parietes of the cavity in which the tumour was situated were successively touched with the cautery, and the posterior was more particularly attended to. The lips of the wound in the cheek were not united by sutures, in order to attack the disease more readily if it should soon re-appear. At the end of a month this unfortunate, but not unexpected event occurred, and fungous granulations shot up from the posterior wall of the sinus. They were destroyed, but without effect, for the disease returned, and the patient died. On examination of the body, the medullary disease was continued along the sheath of the posterior palatine nerves, and the spheno-palatine ganglion with its filaments as far as the superior maxillary nerve.

This case is well adapted to disprove the idea which some surgeons entertain respecting the powers of the actual cautery. As M. Delpech well observes, the readers of the works of Dessault form a very erroneous notion of the success attending the treatment of these cases, and hospital surgeons would seem to shrink from publishing the gloomy results of experience. How satirical a sketch might be given of some of the third or fourth rate surgeons in this country. Through the medium of the journals they acquire a smattering of French practice, and continue to embrace opinions which have already been abandoned by the more practical among the French themselves. Their heads are filled with what was done or thought by Monsieur this or Monsieur that, and if they publish a case it is certain to be dressed up in such a mixture of French and English, such a piebald garment of affected minuteness and real want of wholesome knowledge, that the well-informed are sickened and disgusted. Thus we scarcely open an English journal, without reading of the benefits derived by some embryo surgeon, from the actual cautery in malignant tumours of the antrum or elsewhere, whilst the French hospital surgeons, who have used it as a staple article since the days of Dessault, acknowledge that the opinions propagated among their countrymen are not founded in fact. We have no wish to discard the actual cautery from practice in these cases; on the contrary, we know it to be occasionally very useful. But we quarrel with the rage of the younger men of the present day to fly blindly to what is foreign, without considering whether the leading men of the nation that they imitate, are not amused by the folly and half-informed enthusiasm of the imitators.

*Case* 3. A man, aged 60, was attacked with pain in the teeth of the upper jaw on the right side, which were extracted or fell out, and a red bleeding fungus sprung from their sockets. There was distortion of the nose and other displacement of parts produced, but the orbit was unaffected. An operation was performed as in the last case, and the parts were destroyed extensively towards the palatine arch and nasal fossa, and in the course of the vessels and posterior dental nerves. The disease did not return.

M. Delpech attributes his success in this instance to directing his attention in a particular manner to the vessels and posterior part of the sinus, and he reprobates the practice of merely destroying all the diseased surfaces indiscriminately. All *should* be destroyed, but the part from which the disease takes its rise, ought to be especially rooted out.

*Case* 4. A man of good constitution, ætatis 34, experienced violent pain in the upper canine tooth of the right side, and as it became loose, he pulled it out with ease. Hæmorrhage followed, the pain continued, and a red bleeding fungus sprung from the socket into the mouth. Eight months after its commencement he was seen by M. Delpech. The incisor and contiguous molar teeth were loose, the canine fossa was thrown upwards, and the nose was turned a little to the left, but neither the palate, nor the eye, nor the nasal fossa, were affected. By a perpendicular incision from the eye-lid to the lip, and by dissection, the anterior surface of the tumour was exposed. It was covered here only by soft recent bone, which was readily scooped away, and the whole of the tumour exposed. At its upper part it was continuous with a large branch descending from the suborbital nerve, and when this was cut at a sound part a free hæmorrhage ensued. The tumour was removed, and the cautery applied, the edges of the cutaneous wound were brought together, and the patient was perfectly cured.

In other instances appearances are more deceptive, and the operator finds, when engaged in the midst of an operation, that the limits of the disease have far exceeded his expectations. It is in such a case as this that the coolness and self-possession of a really scientific surgeon are pre-eminently conspicuous.

*Case* 5. A woman, 32 years of age, had experienced, after a confinement, severe pain in the canine and bicuspid teeth of the upper jaw, on the right side. The teeth became loose, and a tumour formed in the canine fossa. She was seen by M. Delpech two years after the commencement of the disease. The pain was severe, the tumour rose to the lower lid, was unequal and cartilaginous in consistence, but did not appear to be of any considerable dimensions. The operation was performed as in the former case, but on arriving at the tumour, which was of very firm medullary character, it was found to occupy the whole of the maxillary sinus, and to lead to the pterygoid region. The teeth, and as much as possible of the palatine arch were removed, the tumour was followed out and discovered to extend into the infra-orbital canal, and it was torn away from its connexions. On separating its posterior surface from before the eroded pterygoid processes, a large jet of blood took place from the internal maxillary artery or one of its last branches. Compression by the finger was applied till the operation was completed, when the cautery was used, and the cavity stuffed with pieces of sponge. Much irritative fever followed, hæmorrhage occurred on the fifth day, was stopped by compression, and returned, the patient was worn out, but not by the bleeding, and on the 20th day after the operation she died. On examination of the body, inflammation was seen to have extended along the 5th nerve to the pia mater.

*Case* 6. A young woman experienced pain in the first large molar tooth of the upper jaw on the right side. The gum swelled and the tooth was removed, but the pain continued, and soon a red and painful fungus arose from the socket. M. Delpech augured badly of the nature of the case, and recommended an operation, but the patient felt a natural reluctance, and applied for other advice. From some silly fancy the case was considered syphilitic, and treated by preparations of gold, when *steel* would have been more effectual. The disease rapidly made progress, and in six weeks the tumour was as large as the head of an adult. All the usual melancholy consequences of the increase of the morbid growth were witnessed, and the patient died worn out with hæmorrhage. On examining the head the disease was perceived to have its origin in the neurilema of the nerve of the 5th pair.

The lower jaw is the seat of malignant tumours following the same march as those in the upper. Of this M. Delpech gives only one case.

*Case* 7. A woman, 37 years of age, enfeebled by frequent child-bearing, was seized with violent pain in the large molar teeth on the right side of the lower jaw. Three fell out spontaneously, and the sockets were filled by a red fungus, covered with a solid cicatrix. The alveoli enlarged, then a point of the cicatrix burst and a fungus sprouted up, the sufferings became intense, and when seen by M. Delpech, the tumour extended from the canine tooth to the coronoid process, was an inch and a half above the level of the teeth, and two inches in breadth. Being satisfied of the malignant nature of the tumour, M. Delpech determined to remove it by operation. Having removed the external bony wall, he arrived at a soft medullary mass, encased in bone. Behind, the tumour extended into another narrow cavity, where it seemed to have a firm attachment. When the morbid growth was removed, it was discovered that at this particular point were situated the dental vessels and the nerve. The latter penetrated the fibrous covering of the tumour, and then, spreading out and becoming more dense, was lost in the medullary mass. The actual cautery was applied to the whole of the exposed surface, and still more carefully to the part where the vessels entered the tumour. All went on well, except at one point before and below the base of the coronoid process, where the patient experienced violent pain, and red, fleshy, and exquisitely sensitive granulations arose. Caustics were tried without much benefit, and the bone which surrounded and concealed the part was broken up. Amongst the fragments thus removed, was the little process which surmounts the inferior dental canal, and the submaxillary nerve being thus shewn to be the seat of the patient's sufferings, it was drawn out by a tenaculum, and divided in a sound part. The tumour twice returned, and each time it grew from a pedicle in the course of the inferior maxillary nerve.

This concludes the series of cases brought forward by M. Delpech, and we leave it to those surgeons who have carefully dissected many of these tumours, to decide if he is right or wrong in his opinions respecting their origin. If reasoning on such a subject were allowable, we should say, from analogy, that the disease would most probably arise from more points than one, or at least that in different cases, different tissues would give it birth. Before we conclude we may notice one point in M. Delpech's practice, which deserves attention. In the malignant disease, when he expects that parts may require removal at a period subsequent to the primary operation, he does not unite the whole line of the integuments but keeps them apart, in order to get more readily at the deeper parts. When all that is separable is separated, he pares the edges, now cicatrized, of the integuments, and brings them together, as in the hare-lip operation. Perhaps this may afford an useful hint on some occasions. We have given the foregoing cases, much abbreviated, to our readers, in order that they may judge in a fair and general way of the results of operations on fungous tumours of the jaws. They show how seldom success attends the scalpel of the surgeon, in cases of a really formidable description.

---

